# Spontaneous cerebrospinal fluid leak following a pilates class: a case report

**DOI:** 10.1186/1752-1947-8-456

**Published:** 2014-12-21

**Authors:** James Davis, Irini Yanny, Sukhdev Chatu, Patrick Dubois, Bu Hayee, Nick Moran

**Affiliations:** 13 Department of Gastroenterology, Kings College Hospital, Denmark Hill, London, SE5 9RS UK; 14 Department of Neurology, Kings College Hospital, Denmark Hill, London, SE5 9RS UK

**Keywords:** Cerebrospinal fluid leak, Pilates, Subdural hematoma, Low pressure headache, Epidural blood patch, Connective tissue disorder

## Abstract

**Introduction:**

A spinal cerebrospinal fluid leak is the most common cause of spontaneous intracranial hypotension which is an uncommon but increasingly recognized cause of headache. This article describes the first reported case of pilates being associated with a spontaneous spinal cerebrospinal fluid leak whilst also highlighting the key information about spontaneous cerebrospinal fluid leaks that will be useful to the general clinician.

**Case presentation:**

We present the case of a 42-year-old Caucasian woman who developed a low-pressure headache following a pilates class. A computed tomography scan of her head demonstrated bilateral chronic subdural hematomas and cerebellar descent. Magnetic resonance imaging of her spine revealed the presence of extensive extradural cerebrospinal fluid collections. She responded to conservative management and repeat neuroimaging after symptom resolution revealed no abnormalities.

**Conclusions:**

Awareness and early recognition of spontaneous intracranial hypotension is important to prevent unnecessary investigations and delay in treatment. Pilates may be a risk factor for the development of a spontaneous cerebrospinal fluid leak.

## Introduction

Spontaneous intracranial hypotension (SIH) is an uncommon but increasingly recognized cause of headache with an incidence of approximately one out of 50,000 [1]. The most common cause is a spontaneous spinal leak [1]. This article describes a case of SIH secondary to a spontaneous spinal cerebrospinal fluid (CSF) leak in a patient who attended ‘pilates reformer’ classes. Given the rarity of this condition, this article will also provide key information about SIH and spontaneous CSF leaks that will be useful to the general clinician.

## Case presentation

A 42-year-old Caucasian woman was admitted to our institution with a four-week history of gradually worsening headache after she had attended a ‘pilates reformer’ class. She described the sudden feeling of a ‘pop’ in the left side of her neck during a certain pilates reformer maneuver but there was no head injury. Our patient developed a headache after one hour, which improved when lying flat. She initially presented to primary care where she received treatment (opiate analgesic and muscle relaxant) for suspected trapezius muscle injury and referred head pain. Over a four-week period, our patient underwent a series of physiotherapy sessions with intensive neck manipulation. During this time, our patient had a number of interactions with primary care, in addition to one emergency department attendance after two-and-a-half weeks. There was no relief with simple or opiate analgesics. The severity of the headache had a profound impact on her ability to perform her activities of daily living. There was no prior history of migraine, head or spine trauma, recent travel or the use of any anticoagulants. She had a history of medically refractory ulcerative colitis treated with subtotal colectomy and subsequent ileo-anal pouch anastomosis. Our patient reported some relief of symptoms in response to a nonsteroidal anti-inflammatory drug (NSAID) but experienced an episode of pouchitis and NSAID treatment was terminated.

**Figure 1:**
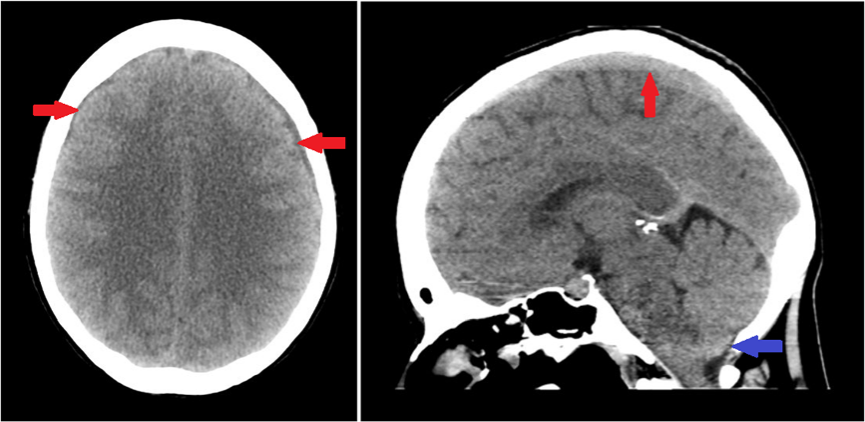
**Non-contrast computed tomography head (axial and saggital sections) showing bilateral chronic subdural hematomas (red arrows), with cerebellar descent (blue arrow) but with no evidence of midline shift.**

**Figure 2:**
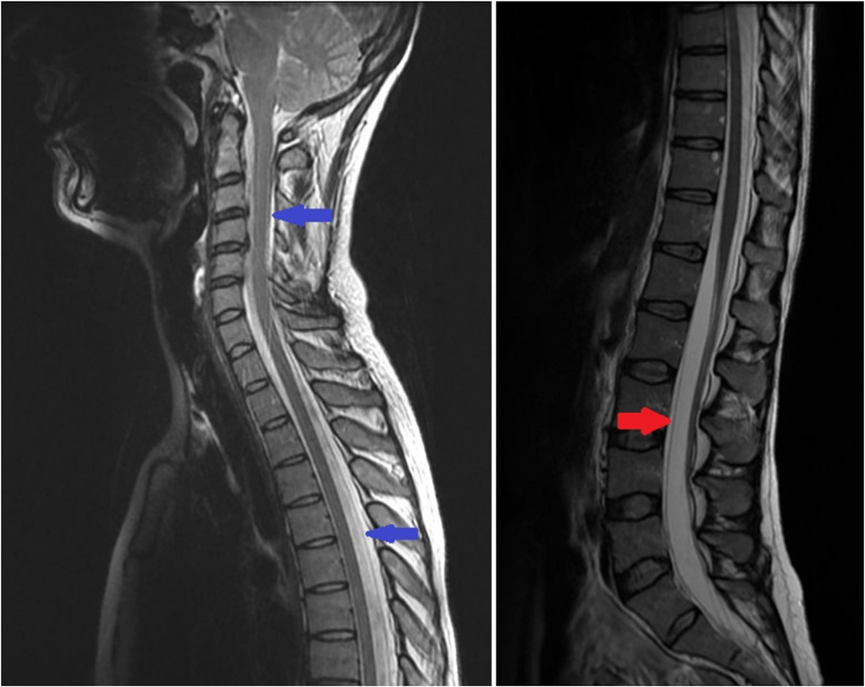
**T2-weighted magnetic resonance imaging (saggital section) showing cerebrospinal fluid collections anterior to the theca at L2-S2 (red arrow) and posterior to the spinal cord at the level of T1-9 and also at C1-2 (blue arrows).** There is no evidence of spinal cord compression and the spinal cord has normal signal.

**Figure 3:**
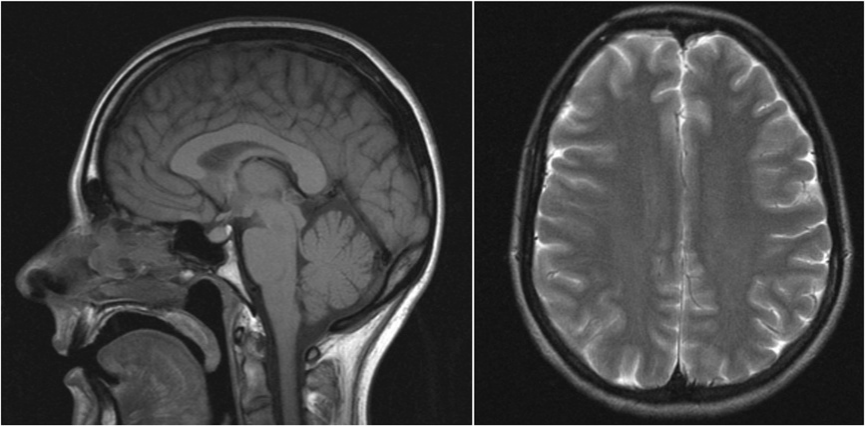
**Intracranial magnetic resonance imaging (axial: T2-weighted and saggital: T1-weighted sections) performed at one month after discharge shows normal intracranial appearances.** The bilateral extra-axial collections and cerebellar descent have resolved.

On admission to our institution she was afebrile with normal blood pressure and pulse. There were no meningitic signs. An examination of the cranial nerves, peripheral nervous system and spine was unremarkable. Basic hematological and biochemical blood tests were unremarkable. A computed tomography (CT) scan of her head demonstrated bilateral chronic subdural hematomas and cerebellar descent (Figure 1). Magnetic resonance imaging (MRI) of her spine revealed the presence of extensive extradural CSF collections (Figure 2) but was unable to identify the exact location of the dural tear. Initially, we opted for conservative management with bed rest and caffeinated drinks rather than use an epidural blood patch. Our patient responded well to the conservative management with a significant improvement in her headaches. She was discharged two weeks after admission and one month after discharge repeat neuroimaging revealed normal intracranial appearances (Figure 3). Our patient was reviewed following this repeat neuroimaging: she remained asymptomatic and, therefore, no further routine follow-up was arranged.

## Discussion

The hallmark symptom of intracranial hypotension is an orthostatic headache that is worse when in the upright position and resolves or improves when lying flat. Other symptoms include diplopia, hearing loss, vertigo and meningism [1].

The most characteristic findings on MRI of the head is pachymeningeal enhancement with downward displacement of the brain, which can be mistaken for a Chiari type I malformation [1]. Subdural hematomas are common in patients with SIH and are usually bilateral but without mass effect [1]. Since the most common cause of SIH is a spontaneous spinal CSF leak, patients should undergo spinal imaging to try and identify a dural tear. CT or MRI myelography are the preferred spinal imaging technique as they provide accurate localization of the tear compared to MRI [1]. Localization of the tear is useful when considering the use of an epidural blood patch; the treatment of choice in patients who have failed conservative management (bed rest, NSAIDs and caffeinated drinks) [1]. Since our patient responded to conservative measures, she did not require an epidural blood patch. There was, therefore, no requirement to determine the exact location of the dural tear using myelography. Clinical improvement usually precedes resolution of the abnormalities documented on neuroimaging of the head although the time between symptomatic and MRI resolution is variable [1].

The underlying cause of spontaneous CSF leaks remains unknown but there is an association with connective tissue disorders [1]. Up to 20% of patients have subtle sketetal abnormalities such as those seen in Marfan syndrome (tall stature, joint hypermobility and arachnodactyly) with no other stigmata of the disease. In some of these patients there is abnormal expression of fibrillin metabolism [1]. It has also been reported that in patients with an underlying dural weakness a small traumatic event may be enough to produce a spontaneous CSF leak [1]. In our patient, there was no evidence of a connective tissue disease and we feel that a trivial traumatic event during her pilates class resulted in the development of a dural tear. A retrospective study of 30 patients with CSF hypovolemia describes one case of spontaneous intracranial hypotension following a yoga class [2]. However, there has been no previous documentation in the literature of ‘pilates reformer’ classes being associated with a spontaneous spinal CSF leak and, to our knowledge, this is the first case.

## Conclusions

The most common cause of SIH is a spontaneous spinal CSF leak and MRI findings in these patients include pachymeningeal enhancement, downward displacement of the brain and subdural hematomas. This case highlights the need for increased awareness and early recognition of SIH in order to prevent unnecessary investigations and delay in treatment. Most importantly, this case raises the possibility of pilates being a risk factor for the development of a spontaneous CSF leak.

## Consent

Written informed consent was obtained from the patient for publication of this case report and any accompanying images. A copy of the written consent is available for review by the Editor-in-Chief of this journal.

## Abbreviations

CSF: cerebrospinal fluid
CT: computed tomography
MRI: magnetic resonance imaging
NSAID: nonsteroidal anti-inflammatory drug
SIH: spontaneous intracranial hypotension.
